# A nondestructive testing method for soluble solid content in Korla fragrant pears based on electrical properties and artificial neural network

**DOI:** 10.1002/fsn3.1822

**Published:** 2020-08-12

**Authors:** Haipeng Lan, Zhentao Wang, Hao Niu, Hong Zhang, Yongcheng Zhang, Yurong Tang, Yang Liu

**Affiliations:** ^1^ College of Mechanical Electrification Engineering Tarim University Alaer China

**Keywords:** electrical properties, Korla fragrant pear, neural network, nondestructive test

## Abstract

The detection of soluble solid content in Korla fragrant pear is a destructive and time‐consuming endeavor. In effort to remedy this, a nondestructive testing method based on electrical properties and artificial neural network was established in this study. Specifically, variations of electrical properties (e.g., equivalent parallel capacitance, quality factor, loss factor, equivalent parallel resistance, complex impedance, and equivalent parallel inductance) of Korla fragrant pears with accumulated temperature were tested using a workbench developed by ourselves. After that the characteristic variables of electrical properties were constructed by principal component analysis (PCA). In addition, three models were constructed to predict SSC in Korla fragrant pears based on the characteristic variables: general regression neural network (GRNN), back‐propagation neural network (BPNN), and adaptive network fuzzy inference system (ANFIS). The results indicated that the GRNN model has the best prediction effects of SSC (*R* ^2^ = 0.9743, RMSE = 0.2584), superior to that of the BPNN and ANFIS models. Results facilitate a successful, alternative application for rapid assessment of SSC of the maturation stage Korla fragrant pear.

## Graphical abstract

A Nondestructive Testing Method for Soluble Solids content in Korla Fragrant Pears Based on Electrical Properties and Artificial Neural Network.

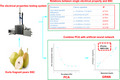

## 1 INTRODUCTION

The Korla fragrant pear is produced in the south of Xinjiang (Wang, Sun, Dong, Zhang, & Niu, 2014) and is one of the most important export fruits from China. Consumer expectations for Korla fragrant pear quality have been steadily increasing over the last decades. The Korla fragrant pear should be nutritious and also should have appropriate texture and taste to meet consumer demands. Soluble solids content (SSC) is one of the most important internal properties because it is a key parameter for determining the overall physical quality and flavor of the pear (Lan, 2017; Nicolaïa et al., 2008; Wang et al., 2014), and it also could provide valuable information for commercial decision‐making (Paz, Sánchez, Pérez‐Marín, Guerrero, & Garrido‐Varo, 2009; Peiris, Dull, Leffler, & Kays, 1999). However, traditionally methods for SSC measurement are mostly destructive(Wang, Zhang, & Ma, 2009), so they just can be applied to small groups of samples, and are not suitable for application in on‐line prediction (Tian, Li, Wang, Fan, & Huang, 2018). Hence, nondestructive sensing of internal SSC in fruit is of great value.

Recently, many studies have reported on multiple nondestructive testing techniques. The techniques based on the electrical properties of fruits and vegetables have attracted widespread attention for the simple and fast operation of the method and its high sensitivity to fruit and vegetable quality. Ates, Ozen, and Carlak (2017) tested the dielectric properties of apples and potatoes using a vector network analyzer (VNA) under the microwave frequency and found that the freshness and degree of aging among the tested foods can be characterized by electrical parameters. Tang, Du, and Zhang (2012) explored the relations of the quality parameters of nectarines with complex impedance, equivalent parallel inductance, and equivalent parallel capacitance under the single frequency of 1MHz and constructed a feasible and effective quality index prediction model. Nyanjage, Wainwright, and Bishop (2001) studied the internal and external epicarp qualities of mangoes after thermal treatment and storage at different temperatures and found that electrical impedance can characterize physiological changes of the fruit. Cortes et al. (2015) analyzed complex impedance during the normal deterioration process of pitayas by using a self‐made double‐needle probe and found that electrical parameters can be applied to evaluate the aging process. These studies demonstrate that electrical parameters can reflect internal quality changes of fruits and vegetables without causing damage to the food. During the growth period of pears, abundant water and electrolytes are concentrated in the fruit. Microscopically, the spatial distribution of many charged particles may change with mutual conversion between substances and energies during the maturation process, thus influencing the macroscopic electrical characteristics of the fruits (Enrico, Aldo, Rudi, Enrico, & Marco, 2013). These results provide the foundations for developing and applying nondestructive tests of SCC in Korla fragrant pears based on electrical properties.

Nelson, Trabelsi, and Kays (2006) have studied the electrical properties of section tissues and correlations of physiological indices of honeydew melon and found a strong linear correlation between electrical properties and SSC. Unfortunately, this method was not overly satisfying when applied to other fruit. No significant linear relations between SSC and electrical properties have been found in peaches (Guo & Chen, 2010), apples (Guo, Zhu, Yue, Liu, & Liu, 2011), Dangshansu pears (Guo, Fang, Dong, & Wang, 2015), Red Bartlett pears (Wang et al., 2009), or other fruits. For this reason, Guo, Shang, Wang, and Zhu (2013) deduced that the traditional linear fitting method might be inapplicable to the prediction of SSC in fruits. Moreover, the relationship between the electrical properties and SSC of Dangshansu pears (Guo, Fang, Liu, & Wang, 2015) during the maturation period was discussed through nonlinear models, such as extreme learning machines (ELM) and GRNN. The prediction results were compared with results of linear models like the multiple linear regression models and the partial least squares regression method, and it was found that the artificial neural network has the best prediction performances. Shang, Gu, and Guo (2015) tested the dielectric properties of nectarines and predicted SSC by using neural network models (e.g., support vector machine (SVM) and ELM) and linear models (partial least squares) and also found that neural network achieved better prediction accuracy. These studies all demonstrate that it is feasible to test and predict fruit quality by combining electrical properties and artificial neural networks. However, to our knowledge, no attempt has been made to predict the SSC of Korla fragrant pears during ripening based on measurement of electrical properties and artificial neural network.

The objective of the present study was to investigate the electrical properties of Korla fragrant pears and develop a artificial neural network model for nondestructive prediction of SSC. Specific aims are (a) to tested electrical properties of Korla fragrant pears based on a self‐made workbench; (b) to used principal component analysis (PCA) construct the characteristic variables of electrical properties; (c) to constructed the prediction models of SSC in Korla fragrant pears based on the characteristic variables: general regression neural network (GRNN), back‐propagation neural network (BPNN), and adaptive network fuzzy inference system (ANFIS); and (d) to determine the optimal artificial neural network model for nondestructive prediction of SSC.

## 2 MATERIALS AND METHODS

### 2.1 Test materials

**Figure 1:**
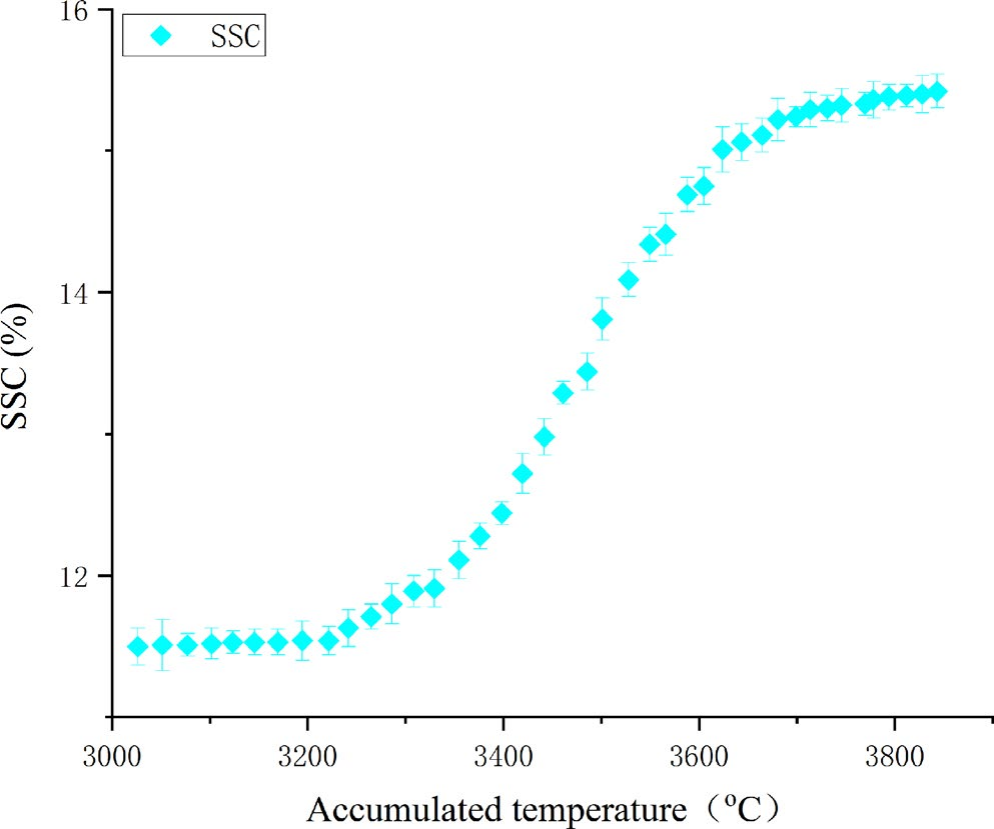
Relationship between soluble solid content and accumulated temperature during sampling

Samples of Korla fragrant pear were collected from the conventional management pear garden in the Alaer City. This is a high‐quality production area of South Xinjiang and the pear trees were twelve years of age. Because the previous study of the research group found that there is a close relationship between SSC and accumulated temperature (Lan, 2017), that during the maturation stage of Korla fragrant pears the accumulated temperature after blossom is lower than 3,000°C and SSC in the Korla fragrant pears is stable. When the accumulated temperature reaches 3,843.5°C, the Korla fragrant pear reached the end of the maturity stage. Based on this, as shown in Figure 1, all samples in this work were picked up in the accumulated temperature lasted from 3,026.50°C after the blossom to 3,843.50°C. (data source: Meteorological Station of Alaer City; corresponding time: August 23–October 2). This accumulated temperature range covers the maturation stage of Korla fragrant pears. Every day, 30 Korla fragrant pears with carpopodium of similar fruit size and color and with no damage or infection were picked in different trees and in different canopy layers to get representative samples which contain a wider range of growth conditions. These samples were processed simply to measure the electrical properties and SSC directly. Then repeat this step the next day.

### 2.2 Test methods

**Figure 2:**
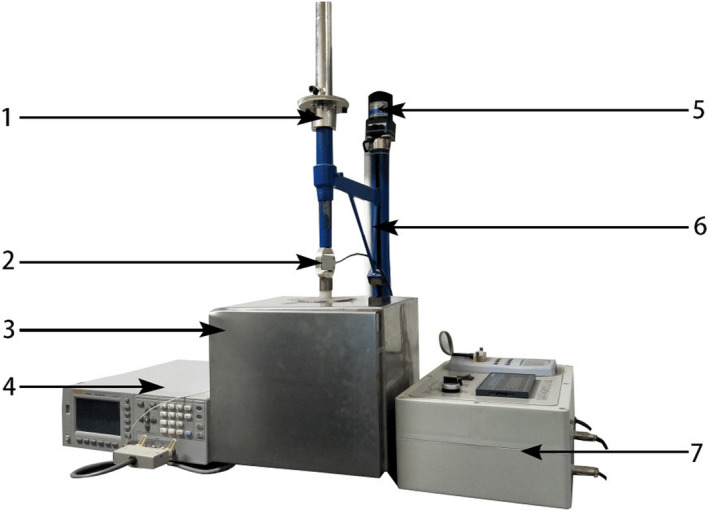
Testing system of electrical properties. 1—fine‐turning hand‐wheel; 2—force sensor; 3—shielded box; 4—testing bridge; 5—loading motor; 6—support; 7—force controller

The electrical properties of the samples were tested by the self‐made testing system in Figure 2. The LCR (inductance, capacitance, resistance) testing bridge was pre‐heated for one hour before use. Subsequently, all electrical properties were set zero to reduce error. Electrodes of the upper parallel‐plate were adjusted slightly by a hand‐wheel to ensure that the electrodes were in close contact with two relative points on the surface equator region of the pears. However, pears are ellipsoid in shape and air in the space between the electrodes and the pear surface is difficult to eliminate. Moreover, air can influence test results significantly. Therefore, conducting resin was coated on the contact surface between the electrodes and pears to eliminate air space completely. Under these conditions, the pear samples were clamped between copper polar plates with a diameter of 10 mm by using 0.5 N tightening force. The polar plates were connected with external mechanical structures by insulating rods and then put in a shielding case with the pears to measure electrical properties, aiming to prevent errors caused by external electromagnetic interference. Before the measurement, wax or impurities on the surface of the pears was cleaned by distilled water and put static in a constant‐temperature (25°C) box for one hour. Equivalent parallel inductance, quality factor, equivalent parallel capacitance, loss factor, equivalent parallel resistance, and complex impedance were tested under the voltage of 100 mV and the frequency of 1 kHz. Pears were also peeled and the juice was collected to test SSC with a portable refractometer (Lan et al., 2015). Mean results were collected after multiple measurements and registered as a percentage value.

### 2.3 Data analysis and processing techniques

#### 2.3.1 General regression neural network

General regression neural network (GRNN) is a radial basis function (RBF) neural network with a highly parallel structure that was developed by Specht (1991). It can approach any function based on nonlinear regression. GRNN has a simple network structure, and it is closely related to training data. As a feedforward network, GRNN is constructed based on training data of a fixed structure and configuration. Different from the traditional neural network, it is easier to optimize parameters of GRNN, without allocation of initial weights. In addition, GRNN adopts single‐pass learning rather than back‐propagation and avoids iterative training. Finally, GRNN converges at the optimized regression surface with the most sample quantity. It also achieves good effect under missing and unstable data due to the high error tolerance and robustness.

#### 2.3.2 Back‐propagation neural network (BPNN)

In the back‐propagation neural network, signals were input through the input layer, calculated in the hidden layer and outputted from the output layer. Next, output signals were compared with the expected output value to calculate the corresponding errors. The network was corrected according to errors from back‐propagation. In this process, threshold and connecting weights among different layers were adjusted by the error gradient descent method (GDM). Each forward propagation layer and the back‐propagation errors were repeated periodically until the output error of the BPNN algorithm was lower than the acceptable level. This is determined by the expected error set by users or the learning period set up by the algorithm. Nevertheless, this algorithm has a major disadvantage for the trend of trapping in a local optimal solution (Sun et al., 2019). This is because the initial threshold and weights are generally initialised in the random number within a certain range.

#### 2.3.3 Adaptive network fuzzy inference system

Adaptive network fuzzy inference system (ANFIS) is a multilayer feedforward neural network based on the Takagi‐Sugeno fuzzy inference system. It combines the neural network learning algorithm and the fuzzy inference system. Based on the advantages of using fuzzy inference systems and artificial neural networks, ANFIS improves the validity of the algorithm in various applications and has the ability to process complicated nonlinear problems. Compared to classical neural networks, ANFIS can be inserted into a network as prior knowledge of the fuzzy rule (for example, if the water is hot, …). The output variables apply fuzzy rules into the fuzzy set of input variables. If the ANFIS model has two inputs (x and y), this results in one output f. For a one‐order Sugeno fuzzy model, the two relevant fuzzy if–then rules are as follows:

(1) Rule 1: If x is A1 and y is B1; then f1=p1x+q1y+r1

(2) Rule 2: If x is A2 and y is B2; then f2=p2x+q2y+r2

where A1, A2 and B1, B2 are fuzzy sets of x and y, respectively. p1, q1, r1 and p2, q2, r2 are parameters of output functions that are determined during the training process. The ANFIS model offers relatively high convergence probability and it can reach a relatively good generalization effect.

### 2.4 Model assessment

To evaluate the prediction accuracy of the models, the prediction performances were compared by root‐mean‐square error (RMSE) and determination coefficient (R^2^). The equation of RMSE is:

(3) $$RMSE=\sqrt{\sum_{i}^{N}\frac{{\left(Y_{E}‐Y_{P}\right)}^{2}}{N}}$$

where Nis the total number of data, $Y_{E}$ is the expected output (test) and $Y_{P}$ is the predicted value of the given input model. Generally speaking, a stable model shall have relatively low RMSE and high *R* ^2^. Specifically, an *R* ^2^ in the range of 0.82–0.90 indicates good performance of the model, while an *R* ^2^ higher than 0.90 is viewed as sufficient to apply to a specific prediction goal.

## 3 RESULTS AND DISCUSSIONS

### 3.1 Analysis of the electrical parameters of Korla fragrant pears

**Figure 3:**
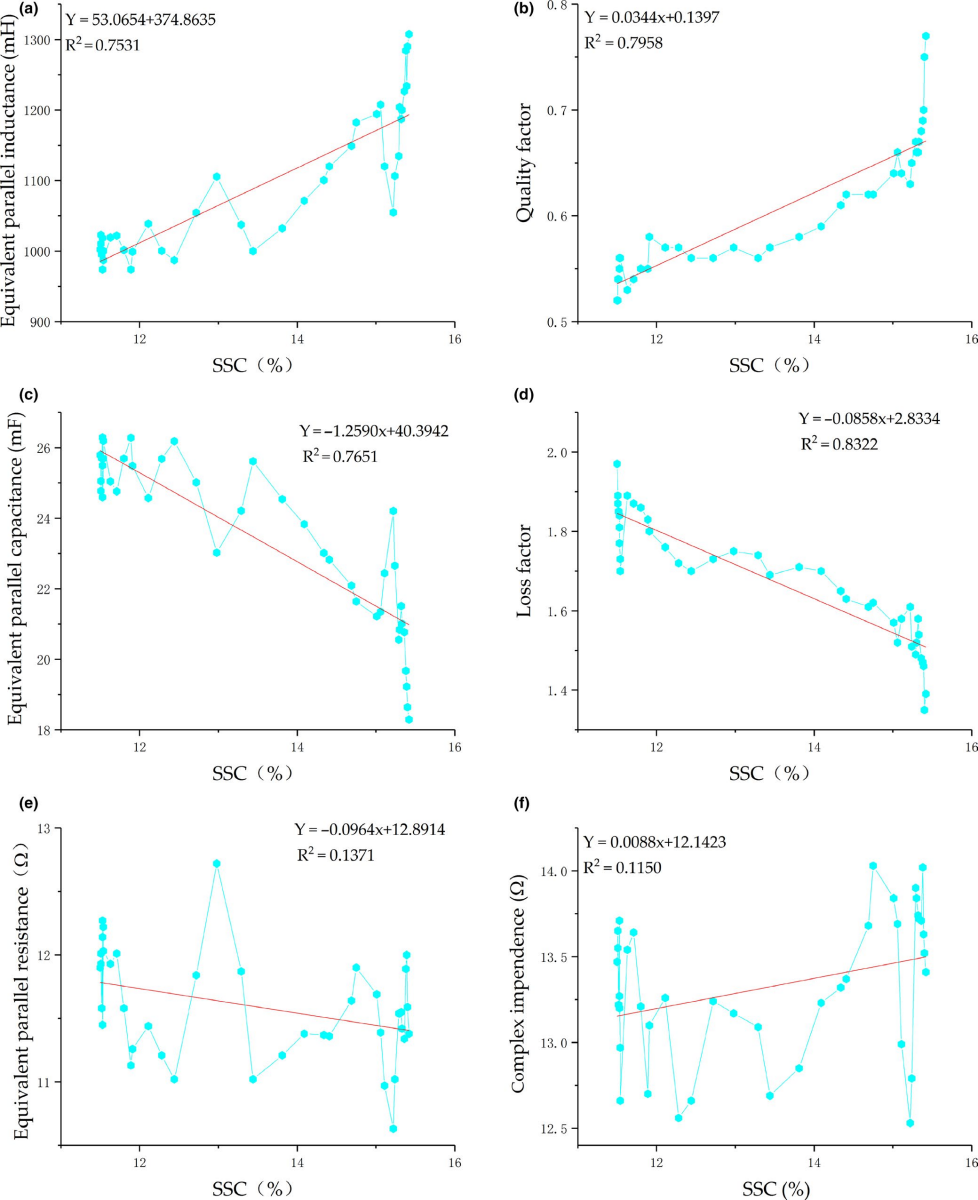
Relations between electrical properties and SSC

The relation between electrical parameters and SSC is shown in Figure 3. Evidently, equivalent parallel inductance and the quality factor of pears increased in fluctuation with the increase of SSC. According to the linear fitting of equivalent parallel inductance and quality factor with SSC, the *R* ^2^ values are 0.7531 and 0.7958, respectively. With the continuous increase of SSC in pears, equivalent parallel capacitance and loss factor dropped in the fluctuation manner and the relevant *R* ^2^ was 0.7651 and 0.8322, respectively. According to the analysis of equivalent parallel resistance and complex impedance, data fluctuated dramatically and showed no significant linear relations with SSC: the *R* ^2^ values are 0.1371 and 0.115, respectively. Based on the above analysis, there is no strong linear relationship between a single electrical property and SSC. The correlation coefficient (*R* ^2^) is less than 0.90. This was similar to the research findings by Guo Wenchuan on peaches (Guo & Chen, 2010), apples (Guo et al., 2011), and Dangshansu pears (Guo, Shang, Zhu, & Nelson, 2015). The results demonstrate that equivalent parallel inductance, quality factor, equivalent parallel capacitance, and loss factor can represent changes in the SSC of pears to some extent. However, it is difficult to predict the SSC of Korla fragrant pears accurately with single electrical properties due to the low determination coefficient. Therefore, it is necessary to predict the SSC of pears through multiple electrical properties and even all electrical properties at once. For this reason, the SSC of Korla fragrant pears was tested by combining PCA and artificial neural network.

### 3.2 Construction of characteristic variables of electrical parameters

According to the Pearson correlation analysis of six electrical parameters in Table 1, SSC has extremely significant correlations with equivalent parallel inductance, quality factor, equivalent parallel capacitance, and loss factor and presents significant correlation with complex impedance. All electrical properties have strong correlations except for equivalent parallel resistance. Equivalent parallel resistance only has extremely significant positive correlation with complex impedance, but significant negative correlation with the SSC of pears. On this basis, there are different degrees of correlation and overlapping information among different electrical properties. But mass information may hinder the analysis and it is difficult to characterize internal correlation with quality. Therefore, it is necessary to construct characteristic variables of the electrical properties of Korla fragrant pears to screen electrical properties and explore implicit variables that are hidden in the original variables and are difficult to measure (Guo, Shang, Zhu, & Nelson, 2015).

**Table 1:** Correlation statistics among the electrical properties of Korla fragrant pears

Index	Equivalent parallel inductance	Quality factor	Equivalent parallel capacitance	Loss factor	Equivalent parallel resistance	Complex impedance	SSC
Equivalent parallel inductance	1.000						
Quality factor	0.929**	1.000					
Equivalent parallel capacitance	−0.982**	−0.937**	1.000				
Loss factor	−0.919**	−0.994**	0.895**	1.000			
Equivalent parallel resistance	−0.044	−0.263	−0.050	0.304	1.000		
Complex impedance	0.594**	0.367*	−0.592**	−0.326*	0.424**	1.000	
SSC	0.868**	0.892**	−0.875**	−0.912**	−0.370*	0.339*	1.000

Note

No label reflects insignificant correlation between two variables (*p* > .05).

* Reflects significant correlation between two variables (0.05 > *p*>.01).

** Extremely significant correlation between two variables (*p* < .01).

To determine influences of equivalent parallel inductance, quality factor, equivalent parallel capacitance, loss factor, complex impedance, and equivalent parallel resistance on the SSC of pears, factor analysis on electrical property indices was carried out after the Pearson correlation analysis. To begin with, the feasibility of the factor analysis method was verified by the Bartlett sphericity test and the significance was less than 0.05, indicating that the relevant matrix was a nonidentity matrix and applicable for factor analysis. Subsequently, a Kaiser–Meyer–Olkin (KMO) test was implemented. The results are presented in Table 2 with the KMO valued at 0.819 (it is generally required to be higher than 0.7), which indicates strong partial correlations among variables. This means the factor analysis effect is relatively good.

**Table 2:** Correlation test between KMO and Bartlett

Kaiser–Meyer–Olkin test	0.819
Bartlett sphericity test	
Bartlett sphericity test	364.649
Degree of freedom	15
Significance	0.000

**Figure 4:**
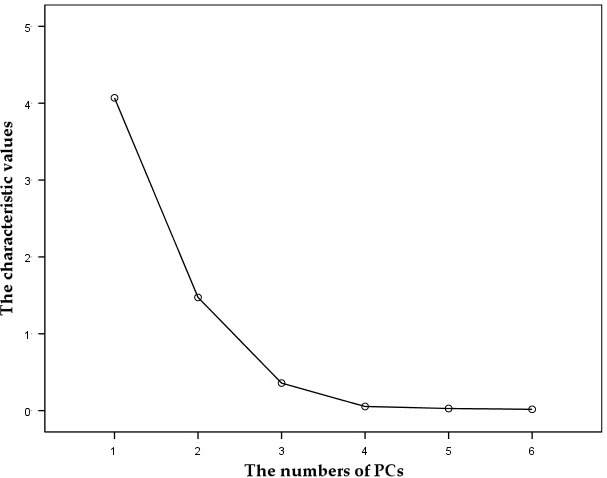
Distribution of the characteristic values of principal components

**Figure 5:**
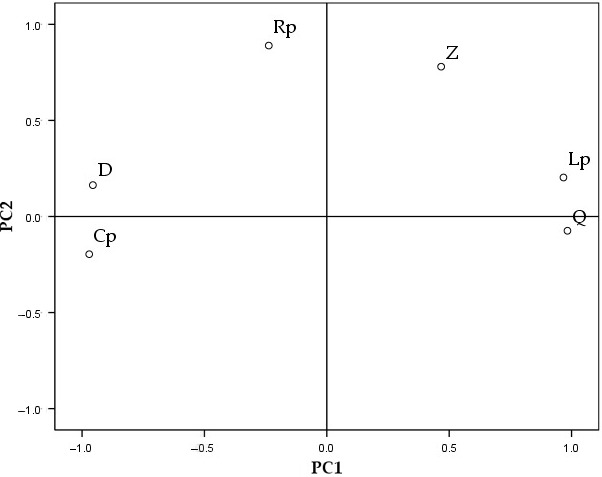
Factor loading after rotation. *Notes*: Cp, Equivalent parallel capacitance; D, Loss factor; Lp, Equivalent parallel inductance; PC1, Principal component "1"; PC2, Principal component "2"; Q, Quality factor; Rp, Equivalent parallel resistance; Z, Complex impendence

It can be seen from Figure 4 and Table 3 that the first two principal components explain 92.386% of the total variance of the original six variables and the characteristic root of these two principal components is higher than one. According to the general principle of determining the number of principal components, the number of characteristics when the characteristic value is higher than one, or the cumulative variance contribution rate is higher than 80%, is equal to the number of principal components. The previous two principal components were chosen in this study. To highlight the electrical properties of the first two principal components, experimental data taken from their loading matrices were processed using the Kaiser standardized tilting rotating method. In other words, the loads of principal components change simultaneously while the weights of the electrical properties remain constant. According to the PCA in Figure 5 and factor loading matrices in Table 4 after rotation, the principal component "1" mainly extracts equivalent parallel capacitance, quality factor, loss factor, and equivalent parallel inductance of Korla fragrant pears, which contributes 67.834% of total variance. The principal component "2" is mainly caused by equivalent parallel resistance and complex impedance in the electrical properties of pears, which contributes 24.553% of total variance. It can be seen from Table 1 that changes of SSC during the harvesting period are mainly manifested by changes of equivalent parallel capacitance, quality factor, loss factor, equivalent parallel inductance, equivalent parallel resistance, and complex impedance.

**Table 3:** Statistics on the distribution of characteristic variables of principal components

Components	Initial characteristic value			Extracted square sum			Rotating square sum		
	Sum	Variance %	Accumulation %	Sum	Variance %	Accumulation %	Sum	Variance %	Accumulation %
1	4.070	67.834	67.834	4.070	67.834	67.834	4.032	67.204	67.204
2	1.473	24.553	92.386	1.473	24.553	92.386	1.511	25.183	92.386
3	0.358	5.969	98.355						
4	0.054	0.896	99.252						
5	0.028	0.464	99.716						
6	0.017	0.284	100.000						

Note

PCA was applied as the extraction method.

**Table 4:** Factor loading matrix after rotation

Indices of electrical properties	Principal components	
	1	2
Equivalent parallel inductance	0.967	0.203
Quality factor	0.983	−0.074
Equivalent parallel capacitance	−0.971	−0.196
Loss factor	−0.956	0.163
Equivalent parallel resistance	−0.237	0.889
Complex impendence	0.467	0.78

Note

PCA was applied as the extraction method. Rotating method: tilting rotating method with Kaiser standardization.

To construct the characteristic variables of electrical properties, the scoring coefficient matrix of different composition variables of the principal components was acquired through regression analysis of the test data. The results are presented in Table 5. Expressions of the top two principal components could be gained from different scoring coefficients (T1 and T2):

(4) $$T_{1}=0.233t_{1}+0.252t_{2}‐0.234t_{3}‐0.249t_{4}‐0.106t_{5}+0.077t_{6}+\delta_{n}$$

(5) $$T_{2}=0.086t_{1}‐0.101t_{2}‐0.082t_{3}+0.159t_{4}+0.61t_{5}+0.5t_{6}+\delta_{n}$$

where $t_{n}$ is the standardized variable of electrical properties: $t_{n}=\frac{\left(x_{n}‐\bar{x_{n}}\right)}{\sigma_{n}}$, $x_{n}$ is variable of an electrical property, $\bar{x_{n}}$ is the mean of $x_{n}$, and $\sigma_{n}$ is the standard deviation of $x_{n}$. $\delta_{n}$ can represent the influencing factors in addition to the variables of the electrical properties and it may be ignored.

**Table 5:** Scoring coefficient matrices of principal components

Indices of electrical properties	Principal component	
	1	2
Equivalent parallel inductance	0.233	0.086
Quality factor	0.252	−0.101
Equivalent parallel capacitance	−0.234	−0.082
Loss factor	−0.249	0.159
Equivalent parallel resistance	−0.106	0.610
Complex impedance	0.077	0.500

### 3.3 Prediction of the SSC in Korla fragrant pears

#### 3.3.1 GRNN prediction of the SSC in Korla fragrant pears

The neural network model chose two principal components that were extracted by PCA method as the network input and SSC as the output. According to 41 groups of datasets, which were acquired by statistical test data, 70% of the experimental data were chosen randomly for training with the GRNN model. In GRNN modeling, the estimated value $\hat{Y}\left(x\right)$ is the weighted term of all observation values of $Y_{i}$. It can be calculated according to:

(6) $$\hat{Y}\left(x\right)=\frac{\sum_{i=1}^{N}Y_{i}\exp\left(‐\frac{D_{i}}{2\sigma^{2}}\right)}{\sum_{i=1}^{N}\exp\left(‐\frac{D_{i}}{2\sigma^{2}}\right)}$$

where $Y_{i}$ is the ith observed value, N is the sample size and σ is the generalized spreading coefficient of Gaussian function, which is called a smooth factor. In this equation, $D_{i}=\sqrt{{\left(x‐x_{i}\right)}^{T}\left(x‐x_{i}\right)}$, where x is the input value and $x_{i}$ is the ith neuron of the corresponding sample.

**Figure 6:**
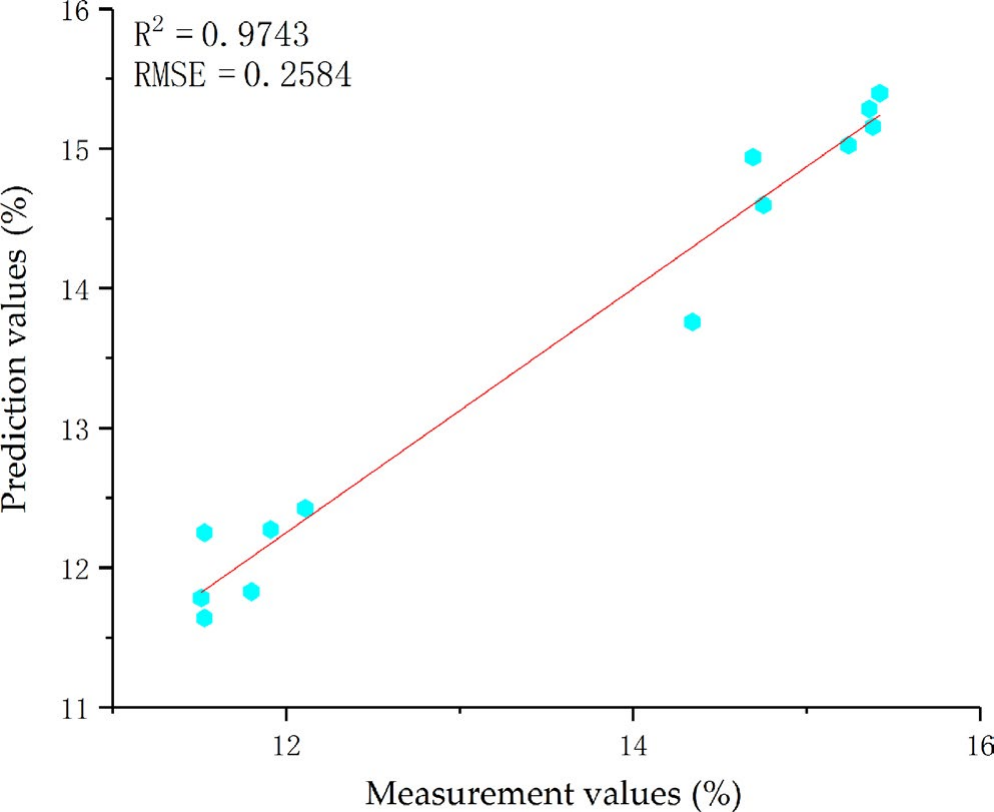
Correlation between prediction values and measurement values of SSC during the verification period of GRNN

As a feedforward network without iterative training, GRNN is constructed by training samples with appropriate smooth factor. The smooth factor σ is used as the only single parameter that GRNN has to learn. It controls the width of RBF and determines the degree of fitting of the regression function. A relatively large or small smooth factor may cause unsatisfying localization results, so it has to determine the optimal value of σ through continuous trial. Based on the examination of output values, it is expected that the best σ of the GRNN model of SSC in pears is 0.1 and the residual 30% of data are input into the trained GRNN model, thus getting a group of predicted values. The linear fitting results between measurement values and prediction values are presented in Figure 6.

#### 3.3.2 BPNN prediction of the SSC in Korla fragrant pears

**Figure 7:**
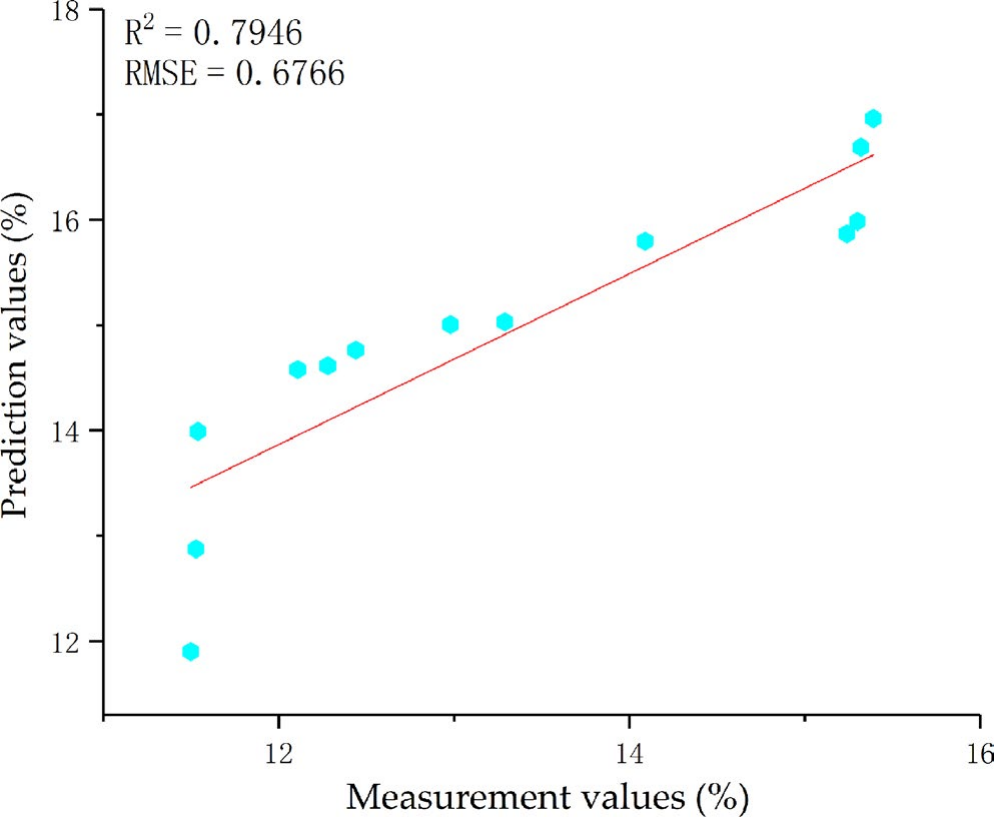
Correlation between the prediction values and measurement values of SSC during the verification period of BPNN

As with GRNN, BPNN analysis used two principal components that were extracted by PCA as the network input and SSC as the network output. Seventy percent experimental data were chosen randomly as the training set, and BPNN was trained for four times by cross‐validation. The number of neurons in the input and output layers was set one, and the number of neurons in the hidden layer was set at 12. The objective mean square error was set at 0.0004, and the learning rate was 0.1. The number of iterations was 1,000. Since the initial threshold and weight of BPNN were chosen randomly within a certain range, the network was trained by 100 times and the network with the best prediction results was used to construct the BPNN. The residual 30% of data were input into the trained BPNN model. The linear fitting results between the measurement values and prediction value of BPNN are shown in Figure 7.

#### 3.3.3 ANFIS prediction of the SSC in Korla fragrant pears

**Figure 8:**
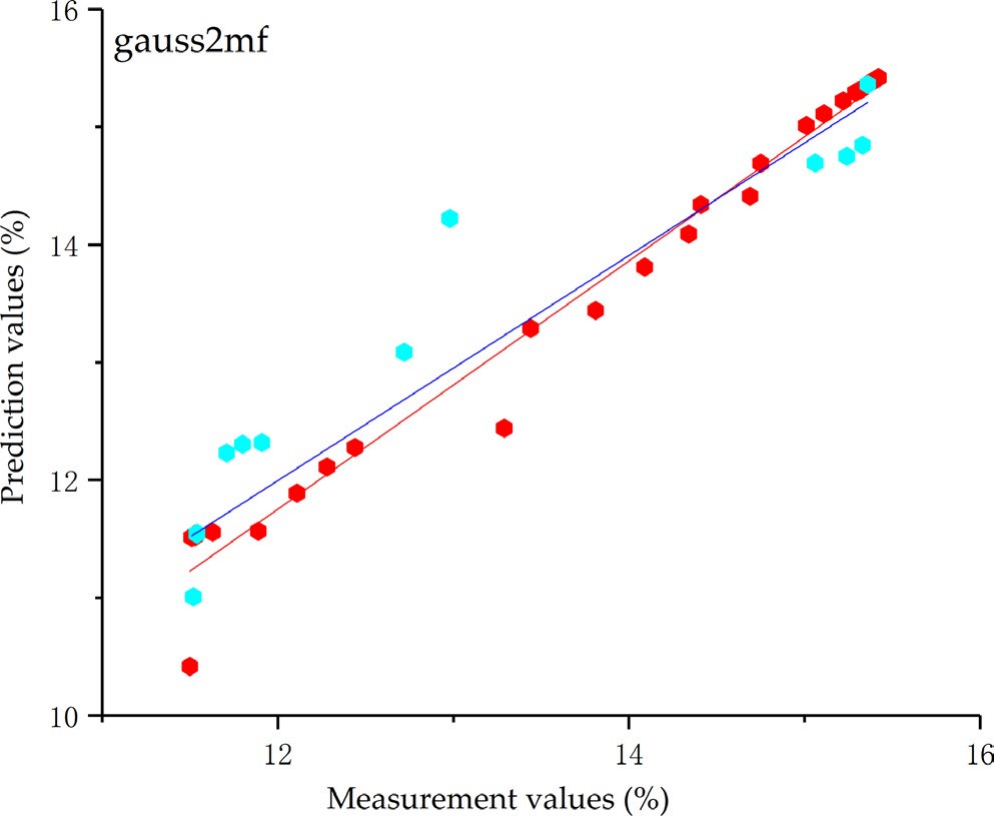
Correlation between the prediction values and measurement values of SSC acquired by gasuss2mf of ANFIS during the training period (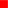) and verification period (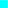)

Experimental data were divided into a training set and verification set according to the proportion of 7:3. Input data were classified by the meshing technique. Meanwhile, the initial ANFIS model, which applied two principal components as the input, was constructed using eight types of MFs for fuzzification of input data. The ANFIS model was tested on an independent dataset after the training stage. Data of the verification set were input into the trained model, thus getting the prediction value. The RMSE and *R* ^2^ of the measurement values and prediction values during the training and verification stages are listed in Table 6. Evidently, the RMSE = 0.7049 and *R* ^2^ = 0.8268 for gasuss2mf during the prediction stage. Compared with the other seven types of membership function, the prediction results of gasuss2mf are stronger. Therefore, gasuss2mf is the most applicable membership function in ANFIS for the SSC prediction. The correlation between prediction values and measurement values is shown in Figure 8.

**Table 6:** Comparison of modeling results of ANFIS membership functions

Membership functions	Training stage		Prediction stage	
	RMSE	R^2^	RMSE	*R* ^2^
trimf	1.5701	0.6495	4.4174	0.6553
trapmf	0.1280	0.9943	2.6684	0.8188
gbellmf	0.2620	0.9835	0.8507	0.8266
gaussmf	0.2083	0.9864	1.1779	0.7131
gasuss2mf	0.2985	0.9783	0.7049	0.8268
pimf	0.2008	0.9861	1.3926	0.725
dsigmf	0.5706	0.9211	1.4079	0.8068
psigmf	0.5706	0.9211	1.4078	0.8068

### 3.4 Prediction results of GRNN, BPNN, and ANFIS

The SSC of the testing samples was predicted by models based on GRNN, BPNN, and ANFIS and the prediction results were compared. The membership function gasuss2mf, which has the best prediction effect of ANFIS, was chosen as the representative. It can be seen from Figures 6 and 7 and Table 6 that GRNN has a higher determination coefficient (*R* ^2^ = 0.9743) and lower RMSE (0.2584) than BPNN and ANFIS, indicating that GRNN is the best model to predict SSC during the maturation stage of pears. The *R* ^2^ of models based on BPNN and ANFIS values 0.7946 and 0.8268 is lower than the linear determination coefficient (*R* ^2^ = 0.8322) between the loss factor and SSC. This demonstrates that the prediction accuracy of these two models was relatively poor, rendering the models inapplicable to predict the SSC of Korla fragrant pears during the maturation stage.

Based on the electrical properties of the Korla fragrant pears during the maturation stage, the nondestructive test of the SSC based on PCA and artificial neural networks was compared with other nondestructive test methods. It was found that GRNN achieved the best prediction performance, manifested by *R* ^2^ = 0.9743 and RMSE = 0.2584. The R^2^ and RMSE of GRNN were slightly higher than the prediction results of Yu, Lu, and Wu (2018) based on deep learning method and hyperspectral technology (*R* ^2^ = 0.890 and RMSE = 1.81), and the prediction results of Tian, Wang, Li, Peng, and Huang (2018) after the fruit characteristic classification based on hyperspectral technology (Rpre = 0.9368 and RMSE = 0.5256). On the other hand, this was similar to the SSC prediction performance of Zhang, Wang, and Ye (2008) based on electronic nose technology (*R* ^2^ = 0.94). This indicates that similar to the hyperspectral technology and electronic nose technology, the nondestructive testing of SSC based on the electrical properties of Korla fragrant pears is a very effective method.

The measurement of electrical properties is simple, but its value is dependent upon being able to relate electrical measurements to physiological or physical properties of mature fruit (Nyanjage et al., 2001). This work demonstrated that it is possible to apply artificial neural network method together with electrical properties measurement technique for predicting maturation stage SSC of Korla fragrant pear. The results obtained by the PCA‐ANN would encourage more research efforts on using electrical properties measurement technique as a novel chemometrical method for the quality detection of fruit in the maturation stage. However, the SSC nondestructive testing method established in this paper is only applicable to the mature stage of Korla fragrant pear, but Yu et al. (2018) study showed that SSC also changed during the storage period of Korla fragrant pear. In addition, researchers have established SSC nondestructive testing methods for fruit maturation stage or storage period at present (Guo, Shang, et al., 2015), but SSC nondestructive testing methods for the whole life cycle of fruit from picking, storage, and selling are not available. Therefore, further research will be conducted on SSC nondestructive testing for the commodity life cycle of Korla fragrant pear.

## 4 CONCLUSION

In this study, we measured the electrical properties of Korla fragrant pears at the maturation stage using a workbench developed by ourselves. The SSC presents poor linear correlation with equivalent parallel capacitance, quality factor, loss factor, equivalent parallel resistance, complex impedance, and equivalent parallel inductance. Single electrical properties are difficult to use in measuring the SSC of Korla fragrant pears. So the characteristic variables of electrical properties were constructed by principal component analysis (PCA). Then, some prediction models of the SSC of Korla fragrant pears during the maturation stage are constructed by combining three artificial neural networks (GRNN, BPNN, and ANFIS). Prediction values were compared and it was found that GRNN is superior to BPNN and ANFIS in predicting the variation trends of the SSC of pears (*R* ^2^ = 0.9743 and RMSE = 0.2584): ANFIS is the second‐best model for SSC prediction (*R* ^2^ = 0.8268 and RMSE = 0.7049). The results of this research prove that it is feasible to predict the SSC of Korla fragrant pears during the maturation stage by combining electrical properties and artificial neural networks. Moreover, this provides a new method for nondestructive testing of the SSC of Korla fragrant pears.

## CONFLICT OF INTEREST

The authors have declared that there is no conflict of interest to this work.

## ETHICAL APPROVAL

This study does not involve any human or animal testing.
